# Coronavirus Disease 2019: A Brief Review of the Clinical Manifestations and Pathogenesis to the Novel Management Approaches and Treatments 

**DOI:** 10.3389/fonc.2020.572329

**Published:** 2020-10-29

**Authors:** Omid Kooshkaki, Afshin Derakhshani, Andelé Marie Conradie, Nima Hemmat, Savio George Barreto, Amir Baghbanzadeh, Pankaj Kumar Singh, Hossein Safarpour, Zahra Asadzadeh, Souzan Najafi, Oronzo Brunetti, Vito Racanelli, Nicola Silvestris, Behzad Baradaran

**Affiliations:** ^1^ Student Research Committee, Birjand University of Medical Sciences, Birjand, Iran; ^2^ Department of Immunology, Birjand University of Medical Sciences, Birjand, Iran; ^3^ Immunology Research Center, Tabriz University of Medical Sciences, Tabriz, Iran; ^4^ Medical Oncology Unit, IRCCS Istituto Tumori “Giovanni Paolo II” of Bari, Bari, Italy; ^5^ Institute of Virology, Freie Universität Berlin, Berlin, Germany; ^6^ Division of Surgery and Perioperative Medicine, Flinders Medical Centre, Adelaide, SA, Australia; ^7^ College of Medicine and Public Health, Flinders University, Adelaide, SA, Australia; ^8^ Department of Radiation Oncology, Mayo Clinic, Jacksonville, FL, United States; ^9^ Cellularand Molecular Research Center, Birjand University of Medical Sciences, Birjand, Iran; ^10^ Department of Biomedical Sciences and Human Oncology, University of Bari “AldoMoro”, Bari, Italy; ^11^ Department of Immunology, Faculty of Medicine, Tabriz University of Medical Sciences, Tabriz, Iran

**Keywords:** cancer, coronavirus disease 2019, coronavirus disease, severe acute respiratory syndrome coronavirus 2, severe acute respiratory syndrome

## Abstract

The recent outbreak of severe acute respiratory syndrome coronavirus 2 (SARS-CoV-2) or coronavirus disease 2019 (COVID-19) in China, which spread to the rest of the world, led the World Health Organization to classify it as a global pandemic. COVID-19 belongs to the *Bettacoronavirus* genus of the *Coronaviridae* family, and it mainly spreads through the respiratory tract. Studies have now confirmed a human-to-human transmission as the primary pathway of spread. COVID-19 patients with a history of diseases such as respiratory system diseases, immune deficiency, diabetes, cardiovascular disease, and cancer are prone to adverse events (admission to the intensive care unit requiring invasive ventilation or even death). The current focus has been on the development of novel therapeutics, including antivirals, monoclonal antibodies, and vaccines. However, although there is undoubtedly an urgent need to identify effective treatment options against infection with COVID-19, it is equally important to clarify management protocols for the other significant diseases from which these patients may suffer, including cancer. This review summarizes the current evidence regarding the epidemiology, pathogenesis, and management of patients with COVID-19. It also aims to provide the reader with insights into COVID-19 in pregnant patients and those with cancer, outlining necessary precautions relevant to cancer patients. Finally, we provide the available evidence on the latest potent antiviral drugs and vaccines of COVID-19 and the ongoing drug trials.

## Introduction

**Figure 1:**
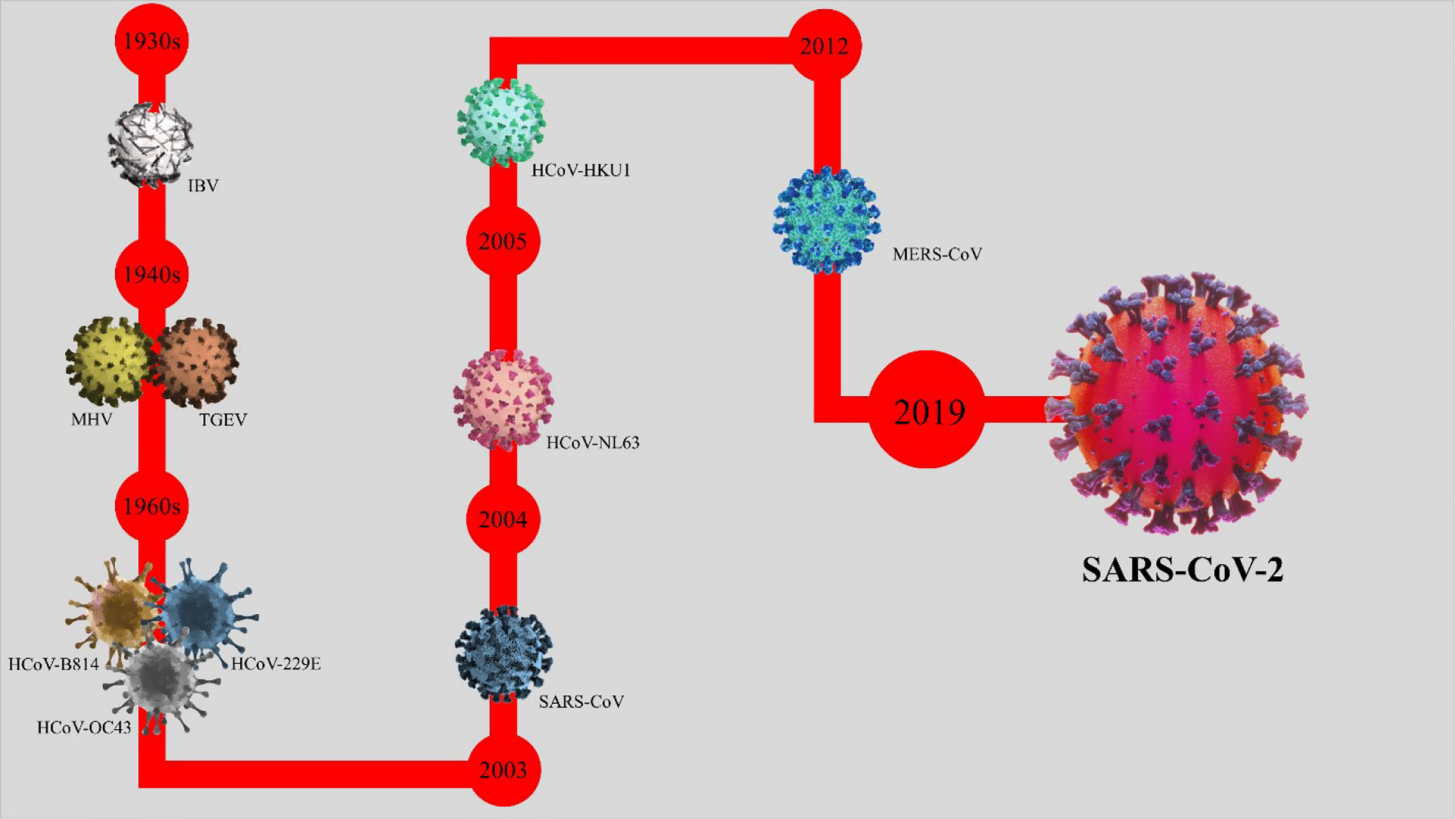
The timeline of the emerging coronavirus. The origin of the history of coronaviruses is pinpointed in the 1930s, when the avian infectious bronchitis (IBV) was first detected in humans. The progression and development of coronaviruses continued to the 1940s (detection of murine hepatitis virus and transmissible gastroenteritis virus), the 1960s (detection of HCoV-B814, HCoV-229E, and HCoV-OC43), 2003 (detection of SARS-CoV or SARS-CoV-1), 2004 (detection of HCoV-NL63), 2005 (detection of HCoV-HKU1), 2012 (detection of MERS), and 2019 (detection of SARS-CoV-2).

Coronaviruses (CoVs) are a family of single-stranded positive-sense RNA viruses that infect humans and many animal species, including bats, civet cats, raccoon dogs, macaques, ferrets, pigs, Himalayan palm civets, livestock, birds, and mice (1–3). The pathologies stemming from CoVs predominantly damage the respiratory, central nervous, intestinal, and hepatic systems (4). CoVs have the largest genome size among RNA viruses and are subdivided into four genera: *Alpha*, *Beta*, *Gamma*, and *Deltacoronavirus*. Until 2003, it was imagined that only two CoVs (HCoV-229E and HCoV-OC43) were responsible for infections of the upper respiratory tract, ranging in respiratory symptoms such as the common cold, pneumonia, and bronchiolitis, but many researchers suspected that there existed more than two HCoVs (5, 6) (**Figure 1**). Unexpectedly, a widespread epidemic of the severe acute respiratory syndrome coronavirus (SARS-CoV-1) broke out between November 2002 and September 2003 that originated in Foshan, Guangdong province, China (7). The SARS-CoV-1 is an extremely pathogenic virus with an overall fatality rate of around 10%, reaching more than 8,000 human infections spanning 29 different countries (8). The SARS-CoV-1 is very contagious and displays influenza-like symptoms, including high fever, myalgia, dyspnea, lymphopenia, and pneumonia (9). The organization of the SARS-CoV-1 genome (29,740 bases long, positive-stranded RNA) is similar to that of other CoVs (10). The World Health Organization (WHO) declared the SARS epidemic to be contained on July 5, 2003, and since then, no SARS outbreaks were reported. Then, in 2012, the Middle East respiratory syndrome-related coronavirus (MERS-CoV) emerged and became the second most pathogenic member of the *Coronaviridae* family in humans in the 21st century. MERS-CoV was first identified in sputum samples of a man in Saudi Arabia admitted with pneumonia (11). Camels were later identified as the cause of spillover to humans and caused the high case-fatality rates in humans across the globe, spanning 27 countries (12). Between 2012 and 2017, WHO reported nearly 2,494 confirmed cases of MERS-CoV infection with at least 858 deaths, the majority from Saudi Arabia. In December 2019, the WHO was informed of cases of pneumonia of unknown cause in Wuhan, China—the third spillover of an animal coronavirus to humans (13). This newly emerged CoV, adopted as SARS-CoV-2 was identified as the cause of a cluster of upper respiratory tract infections or so-called coronavirus disease 2019 (COVID-19), which rapidly emerged in China and rapidly spread to all countries on the globe (13, 14). The WHO declared this rapid escalation of COVID-19 a pandemic in March 2020 and cases continue to rise all over the world, triggering a devastating global health crisis. This review summarizes our current knowledge about the epidemiology, pathogenesis, diagnosis options, and the latest developments in potent antiviral drugs and vaccines for COVID-19.

## Epidemiology

CoVs have been present in humans and various animal species on the planet for hundreds of years, but their exact origin remains unknown. The SARS-CoV-2 belongs to the *Betacoronavirus* genus, and after SARS-CoV-1 (in 2003) and MERS-CoV (in 2015), it is the third major zoonotic coronavirus disease (15). CoVs are a large cluster of single-stranded RNA viruses that can be isolated from different animal species. Phylogenetic and serological studies pinpoint that SARS-CoV-1 and MERS-CoV were a result of a spillover from a wildlife reservoir. Dromedary camels (*Camelus dromedarius*) are now known to be the vertebrate animal reservoir that intermittently transmits the MERS-CoV to humans (16). The outbreak of SARS-CoV-2 originated in Wuhan, China, supposedly from a wildlife market, and the sale of wild animals might be the source of this zoonotic infection (17). The intermediate host for SARS-CoV-2 is currently unknown. Research data suggest that bats (RaTG13) and pangolins carried CoV, thus making them the proximal source of SARS-CoV-2. Accumulating phylogenetic evidence indicates several horseshoe bat species of the genus Rhinolophus within the family Rhinolophidae as a common reservoir for SARS-CoV-1, MERS-CoV, and now SARS-CoV-2 (18, 19). Although RaTG13, sampled from a Rhinolophus affinis bat, is ~96% overall identical to SARS-CoV-2, its spike diverges in the receptor-binding domain (RBD), which suggests that it may not bind efficiently to human angiotensin-converting enzyme 2 (ACE2) (20). Also, evidence from metagenomic sequencing identified various lineages of Malayan pangolins (Manis javanica) coronavirus (Pangolin-CoV) with 92.22% identity to SARS-CoV-2, suggesting pangolins as another possible intermediate host in the outbreak of COVID-19. However, more detailed studies involving pangolin- (Manis javanica) and bat- (Rhinolophus affinis) related CoV (21) are necessary. As of August 5, 2020, a total of 18,445,787 confirmed cases globally with 691,740 deaths had been reported by WHO, and the number is increasing. The United States, with more than 4,850,114 confirmed cases and 159,128 deaths; followed by Brazil (2,808,076 cases, 96,096 deaths); and India (1,908,254 cases, 39,796 deaths) are the three countries with the highest occurrence of COVID-19 (22). Currently, there is no medicine or preventive therapy for COVID-19, and controlling the contagion is the only preventive option to thwart the spread of COVID-19 (23).

## Evolutionary Relationships of SARS-CoV-2

**Figure 2:**
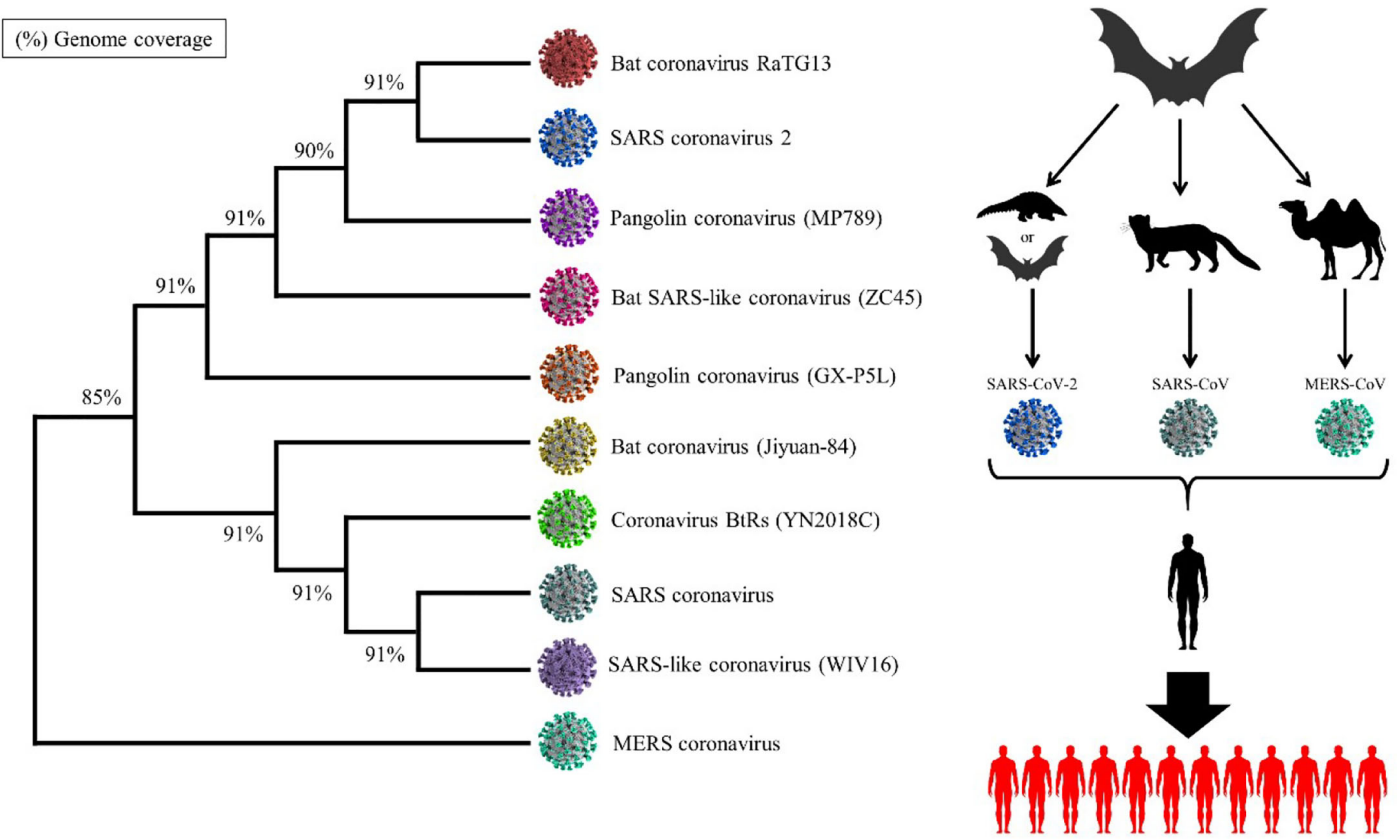
Evolutionary analysis of SARS-CoV-2. The analysis demonstrates that the main similar species to SARS-CoV-2 is bat coronavirus RaTG13.

Sequences most similar to the SARS-CoV-2 cDNA sequence were found using the BLASTn online tool (24). The evolutionary history was derived using the neighbor-joining method (25). The optimal tree with the sum of branch length = 1.11522020 is shown in **Figure 2**. The evolutionary distances were analyzed using the maximum composite likelihood method (26) and are in the units of the number of base substitutions per site. The proportion of sites where at least one unambiguous base is present in at least one sequence for each descendent clade is shown next to each internal node in the tree. This analysis involved 10 nucleotide sequences. Codon positions included were 1st+2nd+3rd+Noncoding. All ambiguous positions were removed for each sequence pair (pairwise deletion option). There was a total of 32,687 positions in the final data set. Evolutionary analyses of SARS-CoV-2 were conducted in MEGA X (27). The final results of the evolutional analysis demonstrated that the coronavirus most similar to SARS-CoV-2 is bat coronavirus RaTG13, and these viruses have the most homology with pangolin coronavirus (MP789). Therefore, it could be concluded that the main origin of SARS-CoV-2 might be due to bats or pangolins. However, the issue of which organism is initially responsible for carrying and transmitting this novel coronavirus to humans has not been thoroughly analyzed.

## Pathogenesis

**Figure 3:**
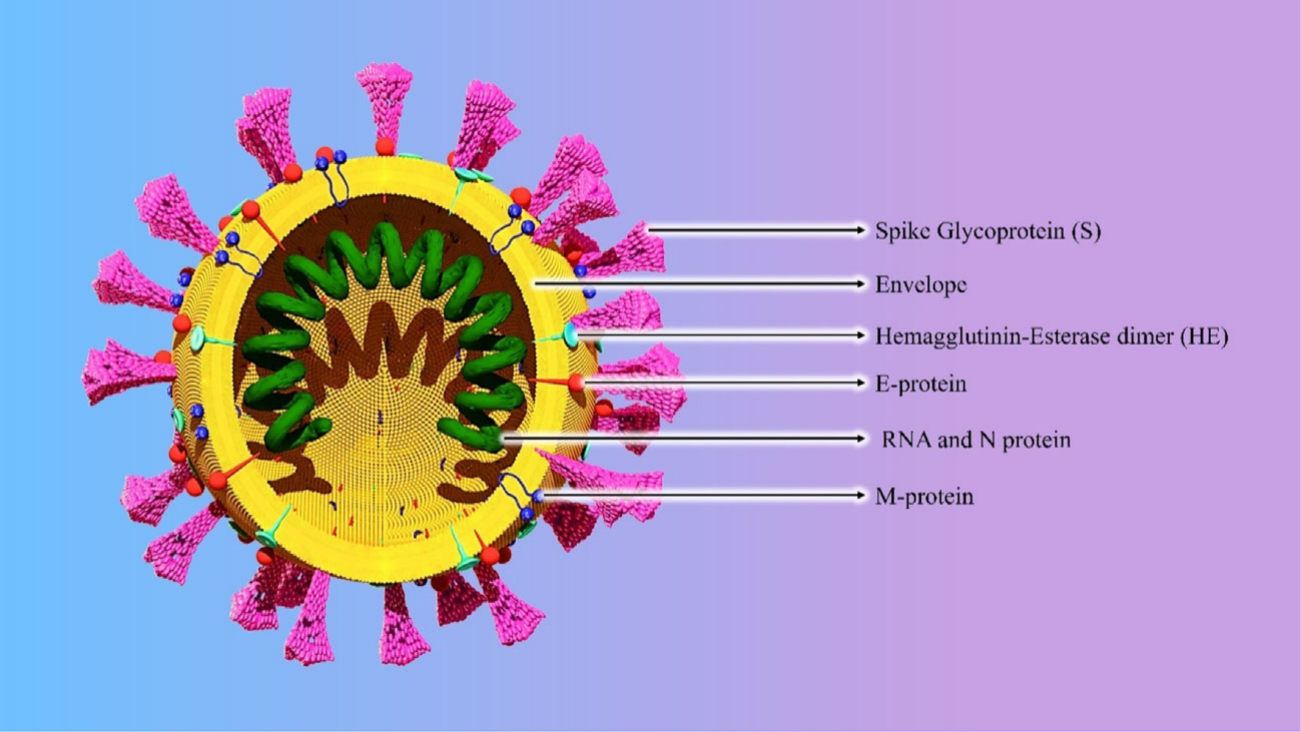
Structure of the SARS-CoV-2.

Recent studies revealed that the genome sequence of SARS-CoV-2 is very similar to that of SARS-CoV-1 and MERS-CoV, therefore serving as models to delineate the pathogenesis of COVID-19 (28). The CoVs encode for several structural and nonstructural proteins required to generate a complete virion. CoVs particles have a variety of packaged host-encoded proteins, including enzymes such as protein kinases cyclophilin A, and apolipoprotein B mRNA editing enzyme, catalytic polypeptide-like 3G (APOBEC3G) that may have critical roles in increasing or inhibiting infection (29). CoVs encode four conserved proteins that are incorporated into the virion: spike (S), envelope (E), membrane (M), and nucleocapsid (N) (30) (**Figure 3**). Studies on SARS-CoV-1 reveal that the S protein, encoded by the first open reading frame (ORF) downstream of the replicase gene, is conserved in all CoVs (31, 32). The S protein is a 180 kDa protein that plays a primary role in attachment and the entry processes in the host organism’s cell. The first step of the infection cycle of enveloped viruses is the interaction with cellular receptors and subsequent fusion with the cell membrane, which is mediated by spike protein (33, 34). The S protein contains an S1 subunit at the N terminus and an S2 subunit at the C terminus; the S1 subunit contains an RBD that engages with the host cell receptor, and the S2 domain contains the membrane fusion machine (30). After the fusion, replication of viral RNA occurs in the host cytoplasm by a unique mechanism in which RNA polymerase binds to a leader sequence and then detaches and reattaches at multiple locations, allowing for the production of a nested set of mRNA molecules with common 3’ ends. The antigen presentation cells (APC) present their antigens as peptides to specific cytotoxic T lymphocytes (CTLs) *via* major histocompatibility complex (MHC, HLA in humans) (35). The antigen presentation of SARS-CoV-1 mainly depends on MHC I molecules, but MHC II also helps its presentation. Subsequently, antigen presentation stimulates humoral- and cellular immunity. IgM and IgG are the main antibodies against the SARS-CoV-1 (36). The IgM antibodies disappear at the end of week 12 although the IgG antibody can last longer. The IgG antibody plays a protective role and is specific for spike and nucleocapsid antigens. A research paper shows that the number of CD4+ and CD8+ T cells and natural killer (NK) cells in the peripheral blood of COVID-19 patients are significantly decreased (37). The reduction of these subsets results in the release of cytokines and pro-inflammatory cytokines (IL-1β, IL-6, TNF-α), IFN-α, IFN-γ, IL-12, IL-18, IL-33, TGF-β, and chemokines (CCL2, CCL3, CCL5, CXCL8, etc.) (38). The continuous production of these cytokines affects NK and CD8 T cells, leading to acute respiratory distress syndrome (ARDS), which is considered the leading cause of death of COVID-19 patients (39).

## Transmission and Symptoms

**Figure 4:**
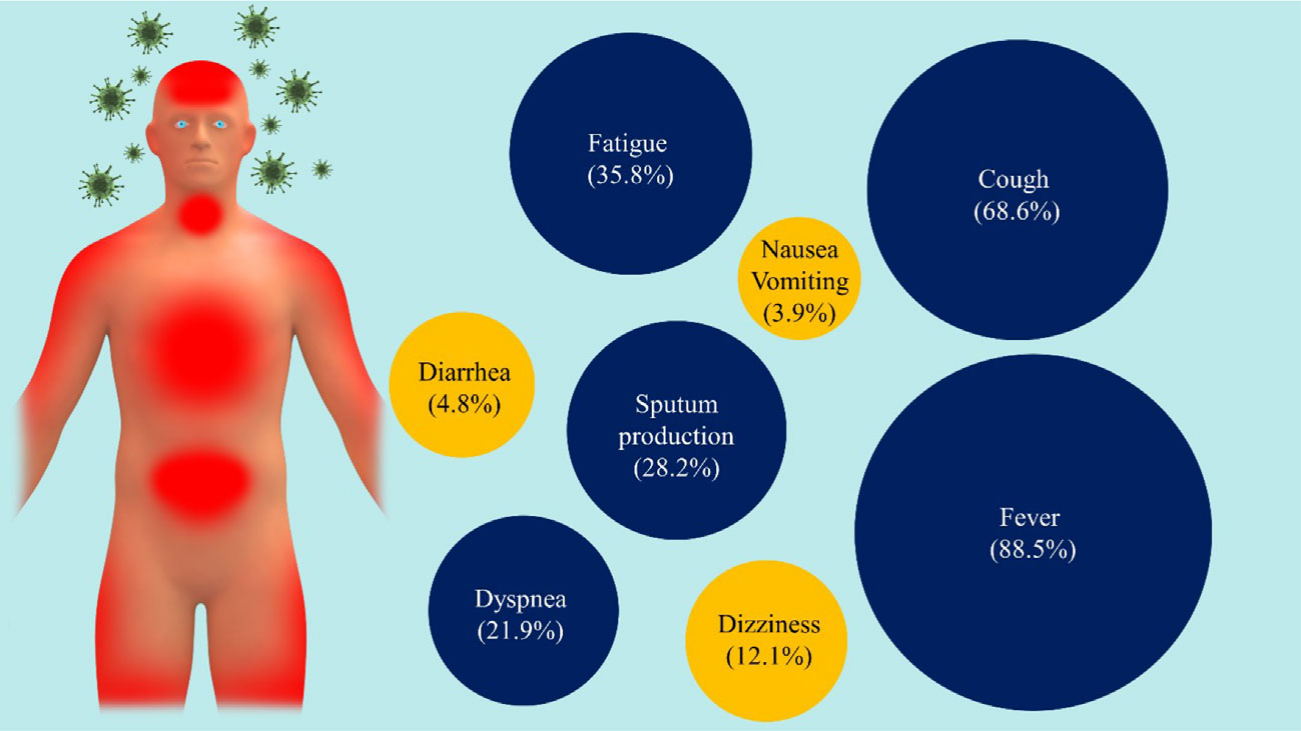
The main symptoms of COVID-19. There are a variety of symptoms reported by people infected by COVID-19 viruses, ranging from mild (yellow) to severe illness (blue).

COVID-19 spreads by human-to-human transmission through droplet or direct contact, but the plausible interspecies mechanism of the virus is not yet fully understood (40). When exposed, the mean incubation period of 6.4 days until clinical signs appear (2020) is confirmed. In models of the transmission process of SARS-CoV-2, the reproduction number (*R*_0_) was predicted to be in the range of 2.24 and 3.58 (the number of new infections estimated to stem from a single case) depending on the exponential growth from the starting date (41). Among patients with pneumonia caused by SARS-CoV-2, fever was the most common symptom, followed by coughing. Although most patients only experienced mild symptoms of the disease, some patients experienced a rapid progression of their symptoms over a week (42). Based on a meta-analysis of 1,994 patients with the COVID-19 infection, Li et al. listed the most common symptoms in order of frequency seen to be fever (88.5%), cough (68.6%), fatigue (35.8%), phlegm or mucus from the throat or lungs (28.2%), and dyspnea (21.9%), and dizziness (12.1%), diarrhea (4.8%), and nausea and vomiting (3.9%) were less common (43) (**Figure 4**). Growing evidence shows that SARS-CoV-2 infection is not limited to the respiratory tract, and other organs may also be involved, including the central nervous system (CNS) causing neurological signs, including headache and nausea (43). Based on the evidence, ACE2 is expressed in the brain, which makes the brain a potential target of COVID-19. In a retrospective case study of 214 hospitalized patients with COVID-19 in Wuhan, China, more than 36.4% of patients were found to have acute cerebrovascular disease, disturbance of consciousness, and skeletal muscle damage as well as neurological manifestations, such as dizziness, headache, nausea, hypogeusia, and hyposmia (44). In another study, Helms et al. enrolled 58 hospitalized patients (median age 63) with COVID-19 and found that 69% of patients had agitation, 67% corticospinal tract signs, and 36% a “dysexecutive” syndrome with difficulty in concentration, attention, orientation, and following commands (45). Specific clinical, diagnostic, and epidemiological studies are needed to help define the manifestations and burden of neurological disease caused by SARS-CoV-2.

## Diagnosis

On routine blood tests, patients may present with an elevated serum C-reactive protein (CRP), erythrocyte sedimentation rate (ESR), lactate dehydrogenase, creatinine, and prothrombin time (46). The clinical diagnosis of COVID-19 is primarily based on real-time quantitative polymerase chain reaction (RT-qPCR) using a nasal swab, tracheal aspirate, or bronchoalveolar lavage samples, and saliva samples. In addition, computed tomography (CT) scan and identification of IgM/IgG by enzyme-linked immunosorbent assay (ELISA) are other important diagnostic tools (40). RT-qPCR is the most popular, useful, and direct method for detecting SARS-CoV-2 in respiratory secretions and blood. Previously, RT-qPCR also presented high sensitivity and specificity for SARS-CoV-1 and MERS-CoV infection (47). Wang et al. examined the sensitivity of the RT-qPCR assay in different tissue samples. Bronchoalveolar lavage fluid specimens showed the highest positive rates at 93% (*n* = 14) followed by sputum at 72% (*n* = 75), nasal swabs at 63% (*n* = 5), fiber bronchoscope brush biopsy at 46% (6/13), pharyngeal swabs at 32% (*n* =126), feces at 29% (*n* = 44), and blood at 1% (*n* = 3) (48). However, RT-qPCR has some limitations, among which are a false-negative rate, difficult nucleic acid detection protocols, and being time-consuming (49). Many physicians proposed using the CT scan of the chest as a primary method because of its high sensitivity (50). In a meta-analysis, Xu et al. evaluated the effectiveness of chest CT for COVID-19 diagnosis. The project included 16 studies for a total of 3,186 patients. They divided the studies into two groups based on the study site (Wuhan and other sites) and found that the patients in Wuhan showed significant sensitivity in the chest CT with the sensitivity values varying from 96% to 99%. In regions other than Wuhan, the sensitivity varied from 61% to 98%. However, the specificity was low (51). In a series of 51 patients (29 men and 22 women) in Taizhou, China, with chest CT and RT-PCR assay performed within 3 days, the sensitivity of CT for COVID-19 infection was 98% compared to the RT-PCR sensitivity of 71% (*p* <.001) (51). Ai et al. compared the diagnostic value of a chest CT scan with RT-qPCR assay in COVID-19 Chinese patients. Although 59% (601 patients) had positive RT-qPCR results, 88% (888 patients) had positive findings on CT scans of the chest with an overall sensitivity of 97% (52). IgM/IgG antibody detection tests are another tool for both diagnosis and patient follow-up (53–55). In addition, asymptomatic cases of COVID-19 showed CT abnormalities. It is difficult to single out and study asymptomatic COVID-19 carriers because people usually get tested for the coronavirus only if they suspect they may have it. Some people who appear asymptomatic at first do later develop classic symptoms, such as high temperatures, fatigue, and difficulty breathing. Long et al. analyzed 37 asymptomatic cases through the contact tracing and testing efforts in Wanzhou, China. The asymptomatic patients were hospitalized for observation. Fifty-seven percent showed lung abnormalities on a CT scan, and others presented with ground-glass opacities: clear signs of inflammation in the lungs. Also, Inui et al. evaluated the chest CT findings in cases from the cruise ship “Diamond Princess” with COVID-19. Of 104 cases, 76 (73%) were asymptomatic, 41 (54%) of which had lung opacities on CT. Another 28 (27%) cases were symptomatic, 22 (79%) of which had abnormal CT findings. Symptomatic cases showed lung opacities and airway abnormalities on CT more frequently than asymptomatic cases (56). It shows that even people with no outward signs of infection could be experiencing some temporary damage to their lungs (57).

The humoral immune system response to SARS-CoV-2 can help to diagnose it. Detecting the production of antibodies, especially IgM, which presents following the infection, can offer an opportunity to enhance detection sensitivity and efficiency. IgM and IgG antibodies appear in the blood at the end of the first week after the manifestation of symptoms (58, 59). Guo et al. evaluated serum IgA, IgM, and IgG responses in COVID-19. In their study, IgA detection showed the highest sensitivity during about 4–25 days after illness onset. The specific IgA reached the peak during 16–20 days after illness onset and then began to decline but remained at a relatively high reading until 31–41 days. The median of RBD-specific IgG was the lowest in early disease stages but increased at 15 days after the onset of the illness, reaching its peak 21–25 days after the beginning of the illness, suggesting that IgG is quite efficient for diagnostics at later stages. Although IgM reached its peak in the early stages, the median of IgM was lower than that of IgA or IgG (60). In both confirmed and suspect cases, the positive rates of IgM antibodies were 75.6% and 93.1%, respectively. When the IgM ELISA assay was combined with qRT-PCR, the sensitivity rate of detection significantly increased (98.6%) (60). Recently, the U.S. Food and Drug Administration (FDA) awarded emergency use authorization (EUA) for Bodysphere’s SARS-CoV-2 IgG/IgM Rapid Test Cassette that diagnoses COVID-19. The IgG/IgM rapid test has a 91% specificity and 99% sensitivity rate and detects antibodies in blood, serum, or plasma in 2 minutes to recognize the current or past history of COVID-19. This test is administered similarly to the blood glucose test (61).

## COVID-19 and Pregnancy

Data on the SARS-CoV-2 in pregnancy are limited, and there is no evidence on transplacental transmission of this virus (62). So far, the clinical symptoms of COVID-19-related pneumonia in pregnant women have been the same as nonpregnant adult patients (63). A total of 12 pregnancies had a mortality rate of 25% in the previous outbreak of SARS-CoV-1, owing to complications such as ARDS (*n*=4), disseminated intravascular coagulation (DIC) (*n*=3), acute renal failure (n=3), pneumonia (*n*=2), and sepsis (*n*=2). In addition, assisted ventilation was three times more likely to be needed among pregnant, compared to nonpregnant, women. Among seven pregnant women that were infected with SARS-CoV-1 in their first trimester, four pregnancies ended in spontaneous abortion. For MERS-CoV, a total of 13 cases reported in pregnant women were published. Two pregnancies were asymptomatic, two pregnancies ended in fetal death, two of them were born premature, and three patients died (64). Two early reports describing 18 pregnancies with COVID-19 showed that all were infected in the third trimester, and clinical findings were the same as nonpregnant adults (65). Fetal distress and preterm delivery were detected in many COVID-19 patients. An intensive review describing 38 pregnant women infected with COVID-19 reveals that, unlike SARS-CoV-1 and MERS-CoV, COVID-19 did not lead to maternal deaths. Similarly to pregnant women infected with SARS-CoV-1 and MERS-CoV, there was no confirmation of the transmission of SARS-CoV-2 from mothers to their fetuses (66). Chen et al. investigated clinical symptoms and possible in utero transmission of SARS-CoV-2 in nine pregnant women. All nine patients had a cesarean section in their third trimester. The reported symptoms included fever (in seven patients), cough (in four patients), myalgia (in three patients), sore throat (in two patients), and malaise (in two patients). Although fetal distress was observed in two cases, none of the mothers developed pneumonia or died. It has been suggested that newborn infants can be infected in other ways and not only in utero transmission. Respiratory viral infections, such as SARS-CoV-2, can occur postpartum through inhalation of the aerosolized agent produced by coughing from the mother, relatives, healthcare workers, or other sources in the hospital (52). It is essential to remember that newborn infants can be infected in other ways than *in utero tr*ansmission, for example, after delivery through the inhalation of the virus aerosols produced by coughing from the infected mother, relatives, or healthcare workers or other sources in the hospital (67). A recent case regarding a 17-day old newborn diagnosed as COVID-19 who developed fever, cough, and vomiting of milk was reported. In this household, the servant was the earliest case; consequently, the mother was infected (68). Shortness of breath, vomiting of milk, cough, and fever are so far the most common symptoms reported in newborn babies (52).

## COVID-19 and Cancer

It has been suggested that individuals older than 60 years and those with a suppressed immune system are especially prone to COVID-19. However, the data on COVID-19 in cancer patients is only beginning to emerge (69, 70). A study from Wuhan reported a higher frequency of COVID-19 in cancer patients as opposed to the general population (0.79% vs. 0.37%) (68), indicating that patients with cancer had a higher risk of severe clinical events than those without cancer. The clinical characteristics of SARS-CoV-2-infected cancer patients include a median age of 65 years with lung cancer being the most common (25%) associated malignancy. The symptoms are similar to other COVID-19 patients, including fever (82.1%), dry cough (81%), dyspnea (50.0%), and lymphopenia (82.1%). The authors of that study opined that cancer patients show poorer outcomes from the COVID-19 and should avoid treatments causing immunosuppression (71). This thought is echoed by other studies given that cancer patients infected with SARS-CoV-2 had a significantly greater risk of needing mechanical ventilation, intensive care unit (ICU) admission, or of dying compared with general patients estimated at 3.5 times greater than the general population (72). This was verified by Liang et al., who evaluated 2,007 COVID-19 patients in a study. They reported a significantly higher mortality rate or ICU admissions in the cases in which the cancer patients required mechanical ventilation (*n*=18) as compared to general patients (39% vs. 8%; *P* = 0.0003) (73). Another important consideration in COVID-19 patients with cancer is the timing of chemo- and radiation therapy and the risks associated with their use. Although cancer treatments may enhance the damaging effects of the virus on the immune system with an increased risk of death and other adverse events (hazard ratio (HR): 4.079, 95% confidence interval (CI): 1.086–15.322, *P* = 0.037) (60), the lack of treatment specifically aimed at cancer increases the risk of death from the malignancy itself. It has been estimated that there is a 16% enhanced risk of death for every month of delay of radiotherapy in patients with head and neck cancer (HR: 1.16, CI: 1.09–1.21) (74). Specific concerns include the use of immune checkpoint inhibitors (ICIs) (75) and confusion in being able to differentiate COVID-19 pneumonia from ICIs-induced pneumonitis. The possible overlap between COVID-19-related pneumonia and antiprogrammed cell death protein 1 (anti-PD-1)/PD-L1-induced pneumonia as one of the adverse events of these agents is the first concern. The overall incidence rate of pneumonitis varies from 2.5% to 5% with PD-1/PD-L1 inhibitors to 7%–10% with a dual combination of Cytotoxic T lymphocyte-associated antigen-4 (CTLA-4)/PD-1 inhibitors (76). Pneumonitis is the most deadly adverse event associated with PD-1/PD-L1 inhibitors with an estimated 35% of treatment-related deaths (77). The second concern is the possible adverse effect of cytokine storm-related to PD-1 inhibitors on the pathogenesis of COVID-19. As described above, the cytokine storm is a common phenomenon of immune hyperactivation in COVID-19 pathogenesis (78). On the balance, it appears that the risks of treating cancer carry a far greater risk of complications than avoiding them. Temporary delay of anticancer therapy in COVID-19 patients who are on long-term disease control with chemotherapy or ICIs may seem prudent until the availability of more robust data (60). To address this critical issue, institutions must continuously analyze and update their clinical policy for dealing with COVID-19 in cancer patients in the light of emerging evidence in literature (79, 80). Other ancillary actions include reducing unnecessary clinic appointments and shifting to telemedicine to improve communication between patients and healthcare workers (81, 82). In **Table 1**, we summarize several considerations and decisions according to the society of surgical oncology (SSO) opinions.

**Table 1:** Therapeutic considerations of several cancers during the SARS-CoV-2 pandemic.

Type of cancer	Therapeutic considerations or decisions	Reference
Breast cancer	**Ductal Carcinoma in-situ:** Defer surgery for 3–5 months. Treat estrogen receptor (ER) + ductal carcinoma *in situ* (DCIS) with endocrine therapy. **Triple-Negative/HER2+ Invasive:** Use of NAC as treatment, Emergency surgery must be performed	(83)
Colorectal cancer	Defer surgery. Proceed with curative intent surgery for non-metastatic patients, for rectal cancer, NAC plus radiation (5 × 5 Gy)	(84)
Thyroid cancer	A severe form of Graves’ disease that does not respond to therapy should urgently be operated. Patients with severe Goiter should urgently be operated.	(85)
Adrenal cancer	Adrenocortical cancer, Cushing’s syndrome, pheochromocytoma, and paraganglioma that did not respond to therapy should urgently be operated.	
Neuroendocrine tumors	Symptomatic small bowel neuroendocrine tumors and tumors with significant growth or short doubling times should urgently be operated	
Renal cancer	cT1a lesions of renal cancer that are manageable with endoscopic resection should undergo endoscopic management, and cT1b tumors should be respected. Patients completing NAC can stay on chemotherapy	
Peritoneal surface malignancy	The operation of patients with malignant bowel obstruction is possible. Defer CRS/HIPEC (an aggressive combination of surgery and chemotherapy) for low-grade appendiceal mucinous neoplasms. Consider NAC for peritoneal metastases from high-grade appendiceal cancer, gastric cancer, colorectal cancer, high-grade mesothelioma, ovarian cancer, and desmoplastic small round cell tumors.	
Lung cancer	The duration of NAC for lung cancer patients with a low stage and better prognosis can be continued during the pandemic. For patients with postoperative lymph node stage N2 with EGFR gene mutations, oral EGFR tyrosine kinase inhibitor (EGFR-TKI) as NAC treatment may be considered a treatment option. Patients with gene mutations including EGFR, anaplastic lymphoma kinase fusion (ALK), and ROS1 fusion, could benefit from targeted oral drugs during the SARS-CoV-2 pandemic, without combination therapy	(86)
Head and neck cancer	Urgently proceed with surgery in these conditions: HPV-negative HNSCC (especially those with airway concerns). HPV-positive HNSCC with significant disease burden or delay in diagnosis, and recurrent HNSCC. Consider deferring> 30 days in following conditions: Low-risk PTC without metastasis and low-grade salivary carcinoma	(87)
Ovarian cancer	Ovarian cancer patients who underwent chemotherapy are fragile. NAC should be favored even if primary cytoreduction surgery could be envisaged. For patients who must undergo the operation, continue the chemotherapy, and operation is suggested after six cycles of chemotherapy.	(88)
Endometrial cancer	Hysterectomy with bilateral adnexectomy associated with a sentinel lymph node procedure should be chosen. Defer surgery for 1 to 2 months in low-risk endometrial cancers. For high-risk patients combining PET-CT and sentinel lymph node biopsy is favored	(89)
Cervical cancer	Defer diagnostic evaluations for 6–12 months. Early-stage cervical cancer: proceed with the standard of care if it is possible. Defer radical trachelectomy or radical hysterectomy for 6–8 weeks or until the pandemic resolves. In low-risk disease (<2 cm, low-risk histology) consider conization or simple trachelectomy ± sentinel lymph nodes. In a gross visible tumor consider NAC	(90)

## Prevention

To help prevent the spread of COVID-19, hands should be washed often, either with soap and water for 20 seconds or a hand sanitizer that contains at least 60% alcohol. There must be a distance between people, and mouth and nose must be covered with a cloth face cover when around others. Until now, there is no vaccine or particular medicine available against SARS-CoV-2. The only intervention strategy is the control of transmission that the majority of countries have activated. The extensive measures to decrease person-to-person transmission of SARS-CoV-2 include isolation, mandatory quarantine strategies of high-incident cities and countries, social distancing, and community containment measures (91).

## Potential Interventions Strategies for COVID-19

The expectancy for vaccines against SARS-CoV-2 has urged scientists to find alternative methods to control COVID-19. Now, clinical trials are investigating the possible effect of therapeutic agents on COVID-19. Some of them are listed in **Table 2**.

**Table 2:** Selected Ongoing (until March 2020) clinical trials of antiviral drugs, chemotherapy agents, and convalescent plasma transfusion therapy against COVID-19.

Intervention/treatment	Number of participants	Country	Primary outcomes	Secondary outcomes	ClinicalTrials.gov Identifier
hyperimmune plasma	49	Italy	Mortality rate	Time to extubating, length of intensive care unit stays, Viral load, Immune response	NCT04321421
TCM prescriptions	340	China	The disappearance rate of main symptoms, Chest CT absorption	Virus antigen-negative conversion rate, Clinical effective time, The number of severe and critical conversion cases	NCT04306497
Sarilumab	400	United States	Resolution of fever, Percentage of patients reporting each severity	Time to improvement in oxygenation, Mean change in the 6-point ordinal scale	NCT04315298
Tocilizumab Injection	330	Italy	One-month mortality rate	Interleukin-6 level, Lymphocyte count, CRP, PaO2	NCT04317092
Sildenafil citrate tablets	10	China	Rate of disease remission, Rate of entering the critical stage	Rate of no fever, rate of respiratory symptom remission, rate of lung imaging recovery, CRP	NCT04304313
Lopinavir/ritonavir, Hydroxychloroquine sulfate	150	South Korea	Viral load	Time to clinical improvement (TTCI), Percentage of progression to supplemental oxygen requirement	NCT04307693
Darunavir and hydroxychloroquine	3040	Spain	Effectiveness of chemoprophylaxis	The virological clearance rate, The mortality rate	NCT04304053
Remdesivir	400	United States	The proportion of Participants With Normalization of Fever and Oxygen Saturation	The proportion of Participants With Treatment-Emergent Adverse Events	NCT04292899
Convalescent plasma transfusion therapy	20	Mexico	Side effects (within 14 days)	Heart Failure, Pulmonary Edema, Allergic Reaction, Viral load	NCT04333355
Convalescent plasma transfusion therapy	10	Colombia	Viral Load, Change in IgG and IgM	Intensive Care Unit Admission, Length of hospital stay, duration (days) of mechanical ventilation, mortality	NCT04332380
Convalescent plasma transfusion therapy	30	Iran	Mortality changes in days 10 and 30, Changes of CRP, IL-6, TNF-α, and PaO2/FiO2 Ratio	Changes of CD3, CD4, CD8, CD4/CD8 ratio, lymphocyte count	NCT04327349
Convalescent plasma transfusion therapy	115	United States	reduction in oxygen and ventilation support	NA	NCT04333251

### Interferons

New therapeutic interventions will likely require a long lead time for the development of approved drugs. Thus, in light of the dire need and urgency to identify the treatment and control of COVID-2019, a repurposing of IFNs and other approved drugs is a potential option in control of COVID-19 infection. Based on previous studies, SARS-CoV-1 and MERS-CoV are valuable for determining the suitability of IFN-I as a treatment strategy in COVID-19. Pei et al. showed that oral administration of IFN-α inhibited the replication of chicken and human CoVs (92, 93). In addition, Morgenstern B et al. showed that IFN-β in combination with Ribavirin inhibited the replication of SARS in human and animal cell lines (94). In vitro studies have already demonstrated that SARS-CoV-2 has greater sensitivity to IFN-I compared with SARS-CoV-1 studies on SARS-CoV-1 and MERS-CoV (95). The vapor inhalation of IFN-α in conjunction with ribavirin is a guideline in China for the treatment of COVID-19 (96). Treatment with IFN-α or IFN-β at a concentration of 50 international units (IU) per milliliter reduces viral titers by 3.4 logs or over 4 logs, respectively, in Vero cells (97). IFN α and γ, alone or in combination, showed partial efficacy against the animal coronaviruses as well as inhibiting SARS-CoV-1 replication *in vitro*. IFN β had the highest potency, demonstrating prophylactic protection and antiviral potential post-infection (98). Zhou et al. studied 77 COVID-19 patients in Wuhan, China, who were treated with nebulized IFN-α2b, arbidol, or a combination of the two. The result showed that the IFN-α2b therapy significantly reduced the duration of the detectable virus and inflammatory markers, IL-6, and CRP (99). In a mouse model of MERS-CoV infection, although treatment with IFN-β in combination with lopinavir-ritonavir did not significantly reduce lung pathology, it improved pulmonary function (100). Collectively, data suggested that interferons (IFN-α and IFN-β) could be a possible answer against COVID-19 therapy (101).

### Intravenous Gammaglobulin

Intravenous gammaglobulin **(**IVIg) is a pool of IgG prepared from serum and primarily used for patients with antibody deficiencies that might be effective against CoVs (102). During the outbreak of SARS-CoV-1, patients in Singapore used IVIg widely. Initiation of IVIg as adjuvant treatment for COVID-19 pneumonia within 48 hours of admission to the ICU can reduce the use of mechanical ventilation, hospital length of stay, and length of stay in the ICU. The rationale for the use of IVIG in SARS-CoV-2 infection is the modulation of inflammation. Cao et al. evaluated IVIg usage in three SARS-CoV-2 patients in China. All three patients were classified as severe, and all had lymphopenia with elevated inflammatory markers. The patients received IVIg at 0.3–0.4 g/kg/day for 5 days. After 2 days, they all had normalization of temperature, and after 5 days of treatment with IVIg, their respiratory symptoms were alleviated. However, this study has some limitations including the concurrent usage of antivirals in two of the three patients and steroids in one patient and the lack of controls (103). In addition, IVIg reduced the 28-day mortality of patients with severe COVID-19 pneumonia (104). In a case report, Lanza et al. reported the successful outcome of IVIg treatment in a 42-year-old Caucasian woman with COVID-19. IVIg was begun, specifically 450 mL (5 mL/kg) at 36 mL/h x 3 days with premedication with antihistamine and rehydration. The patient was discharged after 15 days with two negative swabs and reduced inflammatory markers, normalization of liver function, and recovery of lung function (105). The IVIg would be more beneficial if the immune IgG antibodies were collected from patients who have recovered from COVID-19 in the same city or the surrounding area to increase the chance of neutralizing the virus (106). However, many patients developed several side effects, including venous thromboembolism, and it is prescription limited (107). Many clinical and *in vitro* studies show that switching from IVIg to subcutaneous immunoglobulin can minimize these adverse events. However, the field lacks strong evidence to support the use of IVIg for the treatment of CoVs, such as SARS-CoV-1, SARS-CoV-2, and MERS (108).

### Abelson Kinases (Abl1 and Abl2) Inhibitors

Potent inhibitors, such as Abelson kinase inhibitors (Abl1 and Abl2), were previously tested against SARS-CoV-1 and MERS-CoV (109). In mammals, there are two Abl kinases—Abl1 and Abl2—that control different cellular processes, such as cell survival and proliferation during development and homeostasis (110). It has been shown that Abl2 is required for effective SARS-CoV-1 and MERS-CoV replication *in vitro*. Recently, Coleman CM et al. found that imatinib, an Abl kinase inhibitor, is a possible inhibitor of both SARS-CoV-1 and MERS. They treated Vero E6 (for SARS-CoV-1 and MERS-CoV), Calu-3 (for SARS-CoV-1), and MRC5 (for MERS-CoV) cells with imatinib for either the first 4 h of infection or 5 h after infection in a dose-dependent manner. They found that the reproduction of these viruses was significantly inhibited after treatment with imatinib (109). Regarding SARS-CoV-2, upon binding of the virus spike protein to ACE2, the action of proteases at the cell membrane and in the endosomal compartment is required to complete the subsequent fusion steps of the virus. Abl1 and Abl2 act on proteases required for the completion of these steps by blocking their activity in SARS-CoV-2 (111). Collectively, the FDA-approved Abl2 inhibitors imatinib and saracatinib should be clinically tested for their antiviral effects in the early stage of SARS-CoV-2 infection, either alone or in combination with current antiviral drugs.

### Antiviral Drugs

Remdesivir (GS-5734), as an adenosine nucleotide analog, was developed by Gilead Sciences as a therapeutic agent for treating RNA-based viruses that maintained global pandemic potentials, including Ebola (EBOV) and the CoVs family viruses exemplified by MERS-CoV and SARS-CoV-1 (112). With the demonstration that remdesivir possessed broad activity against RNA viruses, multiple groups assessed antiviral activity both* in vitro* and *in vivo* in several studies validating its activity against CoVs (113, 114). Warren et al. showed that remdesivir had antiviral activity against MERS-CoV with an IC50 of 340 nM *in vitro* (115). De Wit et al. tested the efficacy and antiviral activity of animal models of MERS-CoV infection (116). Their results showed that the treatment with remdesivir 24 h before inoculation effectively inhibited MERS-CoV replication in respiratory tissues and blocked the formation of lung lesions. In vitro studies on cell cultures showed that the remdesivir has half-maximum sufficient concentrations (EC50s) of 0.069 for SARS and 0.074 μM for MERS (117). It may also be considered for a broader range of CoVs, including SARS-CoV-2. A recent *in vitro* study by Wang et al. assessed the antiviral activity of remdesivir against SARS-CoV-2 using qRT-PCR quantification of viral copy number in infected Vero E6 cells. This study showed an IC50 of 770 nM and an IC90 equal to 1,760 nM (118). Remdesivir is administered *via* an IV injection with an initial dose on day 1 (200 mg in adults, adjusted for body weight in pediatric patients) followed by a daily maintenance dose (100 mg in adults) for up to 10 days. Besides, remdesivir didn’t show any serious side effects. Treatments that were approved by the FDA have been assessed for antiviral activity against the COVID-19 causative agent as well.

Lopinavir (LPV), as a protease inhibitor of human immunodeficiency virus 1 (HIV-1) (119) is used in combination with ritonavir (RTV) to increase the LPV half-life against SARS-CoV-1 infection in patients and tissue culture. Deng L et al. assessed Umifenovir (known as Arbidol) and LPV/RTV treatment for patients with COVID-19 in comparison with LPV/RTV only. The main aim of this study was to evaluate the conversion rate of COVID-19 from the date of diagnosis (day 7, day 14) and assessed whether the pneumonia was progressing or improving by chest CT scan (day 7). After 7 days, the SARS-CoV-2 was not detected for 12 (75%) of 16 patients in the combination group compared with 6 (35%) of 17 in the LPV/RTV group (*p*<0.05). Also, after 2 weeks, 94% of patients in the combination group were cured compared to 52.9% in the LPV/RTV group (120).

Nelfinavir is another protease inhibitor used in the treatment of HIV and AIDS (121). Yamamoto et al. showed that Nelfinavir could significantly inhibit the replication of SARS-CoV-1. Consequently, this antiviral drug could be an option for the treatment of COVID-19 (122, 123).

Ribavirin is a guanosine analog that is another antiviral treatment option against COVID-19. Although Ribavirin mainly gained the FDA approval for the treatment of severe respiratory syncytial virus (RSV) infection in children, it shows activity against several RNA and DNA viruses (124). This drug is used to treat several viral infections, including the hepatitis C virus (HCV) and some viral hemorrhagic fevers. Khalid M et al. investigated the efficacy of treatment with Ribavirin and IFN-α in patients infected with MERS-CoV. A total of six patients were enrolled in this study and treated with Ribavirin and IFN-α. The authors declared that three patients after treatment had successful outcomes, and Ribavirin and IFN-α therapy may have effects in MERS-CoV patients (125).

Favipiravir is another drug under clinical development. Favipiravir is a purine nucleoside analog, which acts as a competitive inhibitor of RNA-dependent RNA polymerase (126). Previously, it was demonstrated that favipiravir is effective against influenza A and B and several agents of viral hemorrhagic fever, such as Ebola (127, 128). An *in vitro* study showed an effective activity of Favipiravir against SARS-CoV-2 (EC50 = 61.88 μM) (129). Cai et al. studied the activity of favipiravir in 80 patients with COVID-19 in China. Patients with mild or moderate COVID-19 were enrolled within 7 days from disease onset. Thirty-five patients were assigned to favipiravir and 45 patients to lopinavir/ritonavir. Patients received favipiravir 1,600 mg orally twice daily on day 1 followed by 600 mg on days 2–14. Therapy continued until viral clearance, up to a maximum of 14 days. They reported a significant reduction in the viral clearance of SARS-CoV-2 in patients treated with favipiravir compared with historical controls treated with lopinavir/ritonavir (4 days versus 11 days). Furthermore, the rate of adverse events in patients receiving favipiravir was significantly lower than lopinavir/ritonavir (11.4% vs. 55.6%; *P < *0.01) (130). Also, in a randomized clinical trial, Chen et al. compared the efficacy and safety of favipiravir versus umifenovir in the treatment of 240 patients with COVID-19. The results showed that the 7-day clinical recovery rate was 55.86% in the umifenovir group and 71.43% in the favipiravir group (*P* = 0.01) (131).

### Chemical Drugs

For decades, hydroxychloroquine (HCQ) and chloroquine were used actively to treat infections with intracellular micro-organisms such as malaria (132). Previously, the positive effect of HCQ as a potent inhibitor of the most CoVs like SARS-CoV-1 was reported in several studies. In addition, chloroquine is recognized to prevent viral infection by increasing the endosomal pH required for virus/cell fusion, therefore interfering with the glycosylation (118). Keyaerts and colleagues evaluated the role of chloroquine in the inhibition of SARS-CoV-1 infection in Vero E6 cells. Their result demonstrated a significant decrease in the IC50 (the half-maximal inhibitory concentration) of Chloroquine for antiviral activity (8.8 ± 1.2 μM) versus its cytostatic activity (261.3 ± 14.5 μM) (133). Barnard, D et al. also examined the antiviral activity of Chloroquine monophosphate and diphosphate in a BALB/C mice model of SARS-CoV-1. They injected** **chloroquine (50 mg/kg) intraperitoneally 4 hours before exposure to the virus. The viral lung titers were 5.4 ± 0.5 to 4.4 ± 1.2 on day 3. In this study, the EC50 of chloroquine, chloroquine monophosphate, and chloroquine diphosphate were 1–4 μM, 4–6 μM, and 3–4 μM, respectively (134). Now, clinical trials investigate the possible effect of this agent against SARS-CoV-2 (135). Consequently, 20 clinical trials started in several hospitals in China. The first results showed a significant effect of chloroquine in the reduction of pneumonia, duration of symptoms, and viral clearance compared with the treatment of the control group. Although chloroquine failed to effectively treat mice infected with the SARS-CoV-1, the inhibitory concentration of the chloroquine for the SARS-CoV-2 was closer to 9 μM. This may suggest that chloroquine could be more effective against SARS-CoV-2 than SARS-CoV-1. Based on *in vitro* evidence and still unpublished clinical experience, the panel recommended a chloroquine phosphate tablet, at a dose of 500 mg twice per day for 10 days, for patients diagnosed as mild, moderate, and severe cases of SARS-CoV-2 pneumonia, provided that there were no contraindications to the drug (136). Also, a study by Yao et al. recommends an HCQ regimen of 400 mg twice a day for the first day followed by 200 mg twice daily for the next 4 days (137). In a recent survey, Wang et al. assessed the antiviral efficacy of five FDA-approved agents including Ribavirin, Penciclovir, Nitazoxanide, Nafamostat, Chloroquine, and 2 antiviral drugs (Remdesivir and Favipiravir) in SARS-CoV-2-infected Vero E6 cells (118). The EC50 of Chloroquine in such Vero E6 cells was 6.90 μM, and the EC50 of Remdesivir was 0.77 μM. They found that Remdesivir and Chloroquine are significantly effective in the control of COVID-19 patients (118). Gautret et al. assessed chloroquine with and without azithromycin and found that the combination achieved a reduction in viral load (138). Huang et al. conducted a clinical study to evaluate the efficacy and safety of Chloroquine in hospitalized patients with COVID-19. A total of 22 patients met the enrollment criteria. Patients were then randomized into two groups: 10 patients were treated with Chloroquine 500 mg orally twice daily for 10 days; 12 patients were treated with Lopinavir/Ritonavir 400/100 mg orally twice daily for 10 days. By Day 14, the incidence rate of lung improvement based on CT imaging from the Chloroquine group was more than double that of the Lopinavir/Ritonavir group (rate ratio 2.21, 95% CI 0.81–6.62). These results suggest that patients treated with Chloroquine appear to recover better and regain their pulmonary function quicker than those treated with Lopinavir/Ritonavir (137). Although the use of HCQ is permitted by the FDA, wide use of HCQ has some side effects, including serious cutaneous adverse reactions, fulminant hepatic failure, and ventricular arrhythmias (139, 140).

### ACE2 Analogs

The ACE2 has been recognized as an important receptor for the SARS-CoV-2, and it has been suggested that inhibition of ACE2 might be used for the treatment of COVID-19 patients. Recently, an international group of researchers in Spain, Canada, Austria, and Sweden used human recombinant soluble ACE2 (hrsACE2) to inhibit the infection of this novel life-threatening coronavirus in a dose-dependent manner (138, 141). The SARS-CoV-2 virus was isolated from a nasopharyngeal sample of a COVID-19 patient infected to Vero-E6 cells (cells used for SARS-CoV-2 isolation) with different titers of the virus. The viral RNA was purified from the cells and examined by qRT-PCR. The results of this study showed that the infection of cells in the presence of hrsACE2 significantly prevented SARS-CoV-2 infections to Vero-E6 cells. The hrsACE-2 was also capable of inhibiting SARS-CoV-2 infections to human capillary and kidney organoids (141).

### Convalescent Plasma Transfusion Therapy

Another potential option is plasma therapy. Plasma provided from the COVID-19 convalescents might make therapeutic support available and can decrease the mortality rate of this disease although official data of efficacy is still under investigation (142). Moreover, a research paper published by Shen C et al. evaluated the effects of convalescent plasma transfusion in the treatment of 5 COVID-19 patients. All five patients received antiviral agents and methylprednisolone at the time of treatment. Patients received convalescent plasma transfusion with the SARS-CoV-2 specific antibody (IgG) binding titer greater than 1:1,000, between 10 and 22 days after admission. Twelve days after the transfusion, viral loads decreased and finally became negative. The IgG and Nab titers increased as well. The ARDS was also resolved in four patients, and three patients were detached from mechanical ventilation within 14 days (143). Currently, several ongoing trials are investigating the safety and efficacy of convalescent plasma transfusion as the treatment for COVID-19.

### Potential Vaccines

Effective vaccines are in the long term necessary to inhibit and control SARS-CoV-2. Several types of vaccines that are based on the S protein have been previously assessed. Previous studies on SARS-CoV-1 suggest numerous vaccines using the full-length S protein of MERS-CoV that could be beneficial against this new pathogenic coronavirus, COVID-19 causative agent. Full-length S protein-based vaccines are classified as viral vectors, DNA-based vaccines, nanoparticle-based vaccines, virus-like particle (VLP), recombinant S protein-based vaccines, and recombinant RBD protein-based vaccines (144). Previous trials to produce vaccines for CoVs that are pathogenic for humans have been aimed at SARS-CoV-1 and MERS-CoV and have been tested in animal models as well. However, no approved vaccine has been introduced that is effective against MERS-CoV so far. Until March 2020, there was one DNA-based vaccine against MERS-CoV, which completed the phase I clinical trials in humans. In this phase of the trial, Modjarrad K et al. considered the safety and immunogenicity of a DNA vaccine against MERS-CoV. A total of 75 participants were enrolled in the study and intramuscularly received dose-escalation of 0·67 mg, 2 mg, or 6 mg GLS-5300 1 mL at baseline, week 4, and week 12. The authors reported no vaccine-associated serious adverse events, and immune responses were detected in more than 85% of participants after 2-time vaccinations, suggesting further development of the GLS-5300 as the anti-MERS-CoV vaccine (145). Adenovirus vectors are widely applied for high-level expression of proteins in mammalian cells and attract increasing attention for their potential use as a live recombinant vaccine and also as a transducing virus for use in gene therapy (146). Currently, four viral vectored vaccines including three adenoviral-vectored (ChAdOx1-MERS, BVRS-GamVac, and adenovirus type 5 vector), and one modified vaccinia virus Ankara (MVA) vectored (MVA-MERS-S) are in progress against MERS-CoV and SARS-CoV-2 (147). Presently, three clinical trials are investigating the safety and immunogenicity of ChAdOx1 against MERS-CoV and SARS-CoV-2 infections as well. The MERS001 study aims to assess the safety and the immune responses of ChAdOx1 vaccination in UK healthy volunteers (NCT03399578). In this phase I trial, a total of 48 participants will be recruited, and five experimental groups will receive a different dosage of the ChAdOx1 vaccine intramuscularly. In a phase I/II randomized trial, the aim is to determine the efficacy, safety, and immunogenicity of ChAdOx1 as the candidate SARS-CoV-2 vaccine (NCT04324606). A total of 510 contributors will be divided into five groups and receive ChAdOx1 (IM) and saline as the placebo. The primary endpoints are the evaluation of the efficacy and safety, and secondary endpoints are the evaluation of the efficacy, safety, tolerability, and reactogenicity signs of ChAdOx1 for 7 and 28 days following vaccination. Currently, 2 clinical trials are investigating the safety and immunogenicity of MVA-MERS-S against MERS-CoV (NCT03615911, NCT04119440). NCT04313127 is designed to investigate the safety, reactogenicity, and immunogenicity of recombinant adenovirus type 5 vector against SARS-CoV-2 infection. A total of 108 participants will be divided into 3 groups (low-dose, middle-dose, and high-dose groups) and receive adenovirus type 5 vector (IM) in a dose-escalating manner. The safety of the adenovirus type 5 vector is the primary endpoint and the safety indexes of lab measures, the immunogenicity indexes of the geometric mean titer of the antibody, are some of the secondary endpoints. The LV-SMENP-DC vaccine is another potential vaccine made by adjusting dendritic cells (DCs) with lentivirus vectors (LVs) expressing SMENP (an engineered synthetic minigene that derives from the conserved domains of the viral structural proteins and a polyprotein protease) of SARS-CoV-2 and immune-modulatory genes. Cytotoxic T lymphocytes (CTLs) will be activated through presenting specific antigens by LV-DCs. A phase I/II study in China will be investigating the safety and efficacy of the LV-SMENP-DC vaccine against the COVID-19 causative agent. The primary endpoint of this study is evaluation of safety and the second endpoint is to evaluate the anti-COVID-19 efficacy of the LV-SMENP DC and antigen-specific cytotoxic T cell vaccines (NCT04276896).

### Candidate Antibodies

Neutralizing antibodies (Nabs) constitute a significant part of protective immunity against viral infection in humans. Monoclonal antibodies (mAbs) work in harmony to target different antigenic domains on the viral surface glycoprotein (148). Recognizing the mAbs becomes the first important step toward a more extensive understanding of the protecting antibody response and improving clinical methods against COVID-19. The efficient treatment options against SARS-CoV-2 can be achieved using specific neutralizing monoclonal antibodies, including anti-SARS-CoV-2 neutralizing mAbs, and anti-ACE2 monoclonal antibodies that can straight prevent any stages of the viral life cycle or the receptor proteins located on the host cell to prevent the virus attachment and entry (149). The SARS-CoV-2 infection begins with the interaction of receptor-binding domains located on the S protein and target receptors on the host cell surface, such as angiotensin-converting enzyme 2 (ACE2) (150). The S protein plays a vital role in coronavirus entry and inducing host immune response (151). Consequently, it has been recognized as a key target to develop effective mAbs against SARS-CoV-2 infection. The S1 subunit of S protein has a receptor-binding motif placed in the RBD interacting with the host cell receptor and mediates the virus attachment. Neutralizing mAbs targeting the N-terminal and C-terminal of such RBD, close to the junction of the S1 and S2 domains (152). Since the emergence of MERS-CoV and SARS-CoV-1 outbreaks, several mAbs were identified that showed encouraging outcomes *in vitro* and *in vivo* that could be possibly useful against SARS-CoV-2. **Table 3** shows potential mAbs targeting MERS-CoV and SARS-CoV-1 as well as their mechanism of action.

**Table 3:** Some of mAbs targeting MERS-CoV and SARS-CoV-1 and their mechanism of action.

Neutralizing antibody	Identification Method	Animal model	Disease	Mechanism of action	Reference
80R	Phage display	Mouse	SARS-CoV-1	Binding to the S1 subunit (amino acid residues 426-492) and blocking the interaction of S1 subunit with ACE2	(153)
CR3014	Phage display	Ferret	SARS-CoV-1	Binding to the amino acid residues 318-510 and 565 with high affinity on S1 subunit, blocking the interaction of S1 subunit with ACE2 *in vitro* and *in vivo*	(154)
S230	EBV transformed B cells	Mouse	SARS_CoV-1	Binding to epitopes overlapping with RBD and blocking the interaction of S1 subunit with ACE2 *in vitro*	(155)
1A9	Phage display	Mouse	SARS_CoV-1	Binding to the Heptad repeat (HR) loops including HR1 and HR2 domain on S2 subunit and blocking the interaction of the S2 subunit	(156)
MERS-4	Phage display	Mouse	MERS-CoV	Binding to the C-terminal of the β5-β6, β6-β7, and β7-β8 loops on the receptor-binding subdomain in RBD and blocking the interaction of S1 subunit with DPP4 *in vitro*	(157, 158)
MCA1	Phage display	Mouse	MERS-CoV	Binding to RBD and blocking the interaction of S1 subunit DPP4 *in vitro* and *in vivo*	(159)
G4	Phage display	Mouse	MERS-CoV	Binding to the glycosylated surface on the S2 subunit *in vitro*.	(160)
CDC2-C2	Phage display	Mouse	MERS-CoV	Blocking the interaction of S1 subunit with DPP4	(161)

## Conclusion

SARS-CoV-2 is similar to the SARS-CoV-1 virus in its pathogenicity, clinical spectrum, and epidemiology. Comparison of the genome sequences of SARS-CoV-2, SARS-CoV-1, and the MERS-CoV show that SARS-CoV-2 has closer sequence identity with SARS-CoV-1 compared to MERS CoV. Although several animals, including pangolin (Manis javanica) and bat (Rhinolophus affinis), have been hypothesized to be a reservoir for COVID-19, no animal reservoir has yet been confirmed. Studies suggest that ACE2 is the main human receptor for COVID-19. The entry and spread of SARS-CoV-2 depend on the viral factors, including S protein and its S1 and S2 subunits, ACE2, viral load, viral titer, and viability of virus. Also, the immune system factors, including genetics (such as HLA genes), age, and gender all lead to whether an individual is vulnerable to the SARS-CoV-2 infection, the duration and severity of the disease, and the reinfection. SARS-CoV-2 appears to be more infectious than SARS-CoV-1 or MERS-CoV based on R0 values calculated at the early stage of the outbreak. The majority of asymptomatic individuals with none or mild symptoms can spread viruses to others, which is extremely challenging for preventing the spread of COVID-19. The symptoms range from dry cough, sore throat, tiredness, body aches, fatigue, nausea, vomiting, diarrhea, breathlessness, pneumonia, ARDS, and multiple organ dysfunction leading to death with case fatality rate ranging from 2% to 3% (162). Until now, there is no vaccine or particular medicine available against SARS.CoV-2. The only intervention strategy is the control of transmission that the majority of countries have deployed. Currently, several potent candidates for medications including Nabs, previously discovered vaccines against SARS-CoV-1 and MERS-CoV, and antiviral drugs against other viruses such as Remdesivir, Favipiravir, and HCQ are under urgent investigation in several countries worldwide. Finally, preventive vaccination is highly recommended in light of the future prevention of emerging coronavirus-related epidemics or pandemics.

## Author Contributions 

OK, the first author of the manuscript, collected the data and wrote the primary version of the manuscript. AD developed the idea of the study and revised the manuscript. AC, SB, HS, PS, and OB provided comments and suggestions to improve the quality of the work. NH and AB drafted the figures and tables. SN and ZA contributed: contributing to the English editing of the manuscript. BB and NS: the corresponding authors of the manuscript, contributing to supporting and also supervising the manuscript. All authors contributed to the article and approved the submitted version.

## Conflict of Interest

The authors declare that the research was conducted in the absence of any commercial or financial relationships that could be construed as a potential conflict of interest.
